# Editorial: Multi-robot systems for space applications

**DOI:** 10.3389/frobt.2023.1253381

**Published:** 2023-07-25

**Authors:** Jorge Pomares, Leonard Felicetti, Damiano Varagnolo

**Affiliations:** ^1^ Department of Physics, Systems Engineering and Signal Theory, University of Alicante, Alicante, Spain; ^2^ School of Aerospace, Transport and Manufacturing, Cranfield University, Cranfield, United Kingdom; ^3^ Department of Engineering Cybernetics, Norwegian University of Science and Technology, Trondheim, Norway; ^4^ Department of Information Engineering, University of Padova, Padova, Italy

**Keywords:** space robotics, collaborative robots, planetary robotics, in-orbit robotics, multi-agent

The utilization of robotic systems in space is currently enabling new mission concepts and applications for both in-orbit operations Papadopoulos et al. (2021) and off-world exploration and exploitation Zarei and Chhabra (2022). Space robots are foreseen as essential for numerous on-orbit operations (e.g., servicing, assembly, and manufacturing), and their utilization in ongoing and under-development missions seems already consolidated or, in any case, achievable in a relatively short time Flores-Abad et al. (2014).

However, the increase in the readiness level of such robotic technology and the enhancement of the space-qualified computational, sensory, and actuation capabilities allows for the proposal of new and more challenging missions utilizing multiple robotic and autonomous systems. Missions that involve the construction of large structures (e.g., for the Research Topic and generation of space-based solar power, for ultra-large telecommunication antennas, or for deep space observation via telescopes larger than the James Webb Space Telescope) will definitely ladder on the possibility given by making numerous and specialized robotic systems interact with each other to build, assemble, and maintain large structures that may not be manageable by individual agents Roa et al. (2017).

For example, new active debris removal missions and/or asteroid redirection missions might involve swarms of small fractionated spacecraft approaching, docking, and eventually pushing the target towards favourable orbits. On the other hand, planetary exploration may benefit from cross-platform interactions, e.g., with drones, hopping systems, and rovers coordinating each other for mapping, analyzing, and exploring Mars or other celestial bodies. Finally, distributed and cooperative robots may enable new *in situ* resource utilization concepts and the construction of infrastructures for an eventual pre-human exploration and/or colonization Schuster et al. (2020).

The utilization of heterogeneous and cooperative robotic systems jointly with the use of artificial intelligence is expected to increase the effectiveness of the use of space resources and boost the level of autonomy in the design and control of new space missions. The goal of this Research Topic is thus to offer a description of the frontiers of multiple cooperative and non-cooperative robot systems operating in space and list the enabling technologies that allow new mission concepts with high levels of autonomy and cooperation in space.

We also note that space sensing and perception are crucial for providing autonomous navigation and control for future planetary missions. Autonomous mapping can be performed using Simultaneous Localization And Mapping (SLAM) methods. In this case, the use of a multi-robot approach allows for an increase in the system’s robustness and provides additional redundancy. In van der Meer et al., a multi-robot solution for lunar exploration is described. This approach, called REALMS, proposes a scalable and adaptable solution using homogeneous and heterogeneous rovers. This multi-robot approach allows for increasing coverage and improves system efficiency by executing the required tasks in parallel.

This work aligns with one of the objectives of NASA’s Artemis program, which involves spearheading a series of missions to locate water ice on the lunar surface and to enable *in situ* resource utilization (stepping stones that enable prolonged stays for astronauts on the Moon). In accordance with the requirements of technological advancements in multi-robot systems for the Artemis mission, the NASA Centennial Challenges Program provided the NASA Space Robotics Challenge Phase 2 (SRCP2). The primary aim of this competition was to actively involve the general public in advancing robot localization, coordination, autonomy, and control technologies for a team of robots exclusively dedicated to *in situ* resource utilization within a simulated lunar environment. Within this competition, Martinez Rocamora et al. describes multi-robot systems and associated autonomous operation methodologies for a heterogeneous team of robots that will cooperate within the lunar environment.

Within the field of on-orbit manipulators, multi-robot control systems present several challenges that require further investigation, including feasibility, closed-loop stability, and robustness. There are indeed major technological challenges in space manipulators and autonomous robotic spacecraft, as missions may, for example, involve the execution of uncertain tasks in an unstructured environment. Space manipulators can even work with unidentified and tumbling debris, and there may be inherent uncertainties in the system and payload parameters. To address these questions in space robotics, Kalaycioglu and De Ruiter proposes a novel approach to non-linear model predictive control that uses the concept of passivity for multi-robot systems. The system considered in this study is composed of a chaser spacecraft, a target payload, and two redundant manipulators. By using this approach, the proposed control scheme ensures closed-loop stability and superior performance to other strategies.

In summary, the articles included in this Research Topic provide a good exposure to Research Topic related to enabling technologies and some space missions and applications that utilize multiple robots and autonomous systems. We expect that these studies will contribute towards the use of space and the proliferation of multiple cooperative planetary and on-orbit robotic systems. These robots will thus likely allow the optimal use of space resources and increase the level of autonomy in the design and control of new space missions.
